# Recent advances in bionic scaffolds for cartilage tissue engineering

**DOI:** 10.3389/fbioe.2025.1625550

**Published:** 2025-07-11

**Authors:** Yushan Zhang, Weihan Yu

**Affiliations:** ^1^ Department of Orthopedics, Xinchang Hospital Affiliated to Wenzhou Medical University, Xinchang, Zhejiang, China; ^2^ Department of Orthopedics, Shanghai General Hospital, Shanghai Jiao Tong University School of Medicine, Shanghai, China

**Keywords:** bionic scaffolds, cartilage, osteoarthritis, regeneration, tissue engineering

## Abstract

Articular cartilage is difficult to regenerate. It often leads to osteoarthritis after injury, which seriously affects the quality of life of patients. Presently, the clinical treatments of articular cartilage injury have certain limitations. With the development of tissue engineering, cartilage repair becomes possible. Different types of bionic scaffolds have shown great application potential in cartilage repair. We reviewed the characteristics of ideal bionic scaffolds, including biocompatibility, biodegradability, mechanical and structural properties, bioactivity and functionality. We also summarized the latest research progress of different bionic scaffolds in recent years, hoping to provide a reference for the design of bionic scaffolds with stable performance and definite efficacy, and help them to be gradually applied in clinical practice.

## 1 Introduction

Osteoarthritis (OA) is a prevalent degenerative disorder influenced by various factors such as aging, overweight, genetic susceptibility, and trauma, with the primary pathological feature being the progressive damage to articular cartilage (Jiang, 2022; Zou et al., 2023). Unlike other tissues, articular cartilage has a unique structure with limited blood vessel, nerve, or lymphatic vessel (Thomas and Mercuri, 2023). Articular cartilage is abundant in extracellular matrix (ECM), which results in low self-repair capacity due to insufficient cells and growth factors (Wu et al., 2020; Wang M. et al., 2022; Guo et al., 2023). Moreover, current clinical treatments, such as microfracture surgery and cartilage transplantation, can alleviate symptoms in the short term but struggle to achieve functional tissue regeneration, leading to the formation of fibrocartilage and facing limitations such as insufficient donors and immune rejection, resulting in poor clinical applicability (Wang et al., 2024a). Thus, there is a growing demand for regenerative strategies to promote cartilage regeneration or replacement.

The rapid evolution of tissue engineering techniques has presented cartilage repair strategies centered on scaffold materials as a promising approach to overcome the long - standing bottlenecks in cartilage regeneration. Notably, several scaffold systems have successfully transitioned from preclinical research to clinical implementation (Klimak et al., 2021).

Scaffolds serve as 3D platforms for cell adhesion, proliferation, and differentiation. They replicate the physicochemical characteristics of the native ECM, modulating the microenvironment for cartilage regeneration through precise control of mechanical properties, degradation kinetics, and the spatiotemporal delivery of bioactive factors. Substantial advancements in osteoarthritic cartilage regeneration can be attributed to the utilization of biomaterial-based scaffolds, which exhibit exceptional capabilities in establishing a permissive 3D milieu that facilitates cell growth and differentiation, thereby offering new therapeutic opportunities for patients afflicted with osteoarthritis (Shalumon and Chen, 2015).

Researchers have harnessed the potential of bioactive molecules, including growth factors or cytokines, into scaffolds to augment *in vivo* regenerative processes. These bioactive entities function as molecular messengers, orchestrating cellular responses that culminate in chondrogenesis and subsequent tissue regeneration.

Concurrently, the provision of mechanical support by scaffolds is imperative for the development of structurally stable and functionally competent cartilage tissue. With the exponential growth of nanotechnology, bio-scaffolds have emerged as highly promising materials in the realm of osteoarthritic cartilage regeneration. Their distinctive capacity to recapitulate the native ECM, establish a conducive 3D environment, and enhance the bioactivity of therapeutic molecules has resulted in their extensive application in regenerative medicine.

Material innovations, ranging from natural polymers to synthetic polymers, composite hydrogels to biomimetic gradient scaffolds, have contributed to the enhancement of cartilage repair outcomes. Nevertheless, the equilibrium between biocompatibility, mechanical strength, and functional orientation persists as a pivotal challenge in contemporary research. This review summarizes recent progress in cartilage tissue engineering, comparing the physical properties and therapeutic effects of scaffolds fabricated with different biomaterials. In addition, this review discusses design strategies, performance optimization, and clinical application prospects of various scaffold materials and explores the mechanisms of action and therapeutic potential of different bionic scaffold materials, hoping to provide theoretical reference and enlightenment for articular cartilage regeneration therapy.

## 2 Ideal bionic scaffolds for cartilage engineering

To effectively accommodate the unique histological characteristics of cartilage, several key aspects must be considered when developing ideal bionic scaffolds.

### 2.1 Biocompatibility and biodegradability

The design of scaffolds cannot be separated from two fundamental considerations: biocompatibility and biodegradability (Lopa et al., 2018). Biocompatibility, defined as the ability of the scaffold to interact with local tissue safely without apparent hazardous effects, is a crucial property that must be considered. Biodegradability is the ability of the scaffold to degrade slowly and be metabolized by enzyme, facilitating the regeneration (Frassica and Grunlan, 2020). Moreover, the degradation products should not induce any degree of cellular toxicity or interfere with the differentiation and proliferation of stem cell or chondrocyte (Williams, 2019). Numerous scaffolds have been designated for cartilage repair, among which, hydrogels made from biodegradable synthetic and natural polymers are of particular interest due to their desired biocompatibility and biodegradability (Shi et al., 2024).

### 2.2 Mechanical and structural properties

The mechanical and structural properties of scaffolds are pivotal to restore cartilage tissue (Rezuş et al., 2021). Ideal scaffolds should offer adequate mechanical stimuli to facilitate cell growth and differentiation (Ngadimin et al., 2021). Cartilage tissue is constantly subjected to diverse mechanical loads, including compression, tension, and shear, during daily physiological activities. Scaffolds should bear appropriate strength and stiffness, which ensures the structural integrity of the repaired site, preventing collapse, deformation, or rupture under mechanical stress (Gilbert et al., 2021). Additionally, the compressive modulus of materials should closely match that of native cartilage. Chondrocytes can maintain a favorable phenotype when the modulus of scaffolds lies within an optimal range. The reported compressive modulus of articular cartilage is 0.02–1.16 MPa in superficial zone and 6.44–7.75 MPa in deep zone (Chen et al., 2001).

Porosity is also important for mechanical properties. Proper porosity facilitates nutrient flow, which affects cell proliferation, migration, and ECM secretion (Wang S. et al., 2022). Furthermore, it can effectively modify the mechanical properties of scaffolds (Cheng et al., 2018). Increasing the pore size or volume fraction can reduce the stiffness of the scaffold and facilitate tissue integration (Ciritsis et al., 2018). If the porosity is insufficient, the available space may be inadequate to support cell migration and proliferation. Conversely, an excessively large porosity can lead to a reduction in mechanical properties, and it can be difficult for the cells to adhere.

In addition, scaffolds should have exhibit long-term mechanical durability. They can resist significant performance degradation under prolonged mechanical stress, guaranteeing the progression of the cartilage-repair. Since scaffolds also need to be degradable, how to ensure the appropriate mechanical durability on the basis of biodegradability is an important issue for the construction of an ideal scaffold.

### 2.3 Bioactivity and functionality

Cartilage is unable to repair itself due to the slow rate of chondrocyte proliferation and regeneration (Lin et al., 2022). In order to accelerate the cartilage repair process, exogenous intervention is necessary. Bionic scaffolds in combination with different interventions, such as implantation of bioactive factors, cells, extracellular vesicles, and drugs, can promote cartilage regeneration (Nordberg et al., 2022). More importantly, smart bionic scaffolds can be designed to have targeted bio-activity and functional characteristics (Fan et al., 2020). By incorporating different growth factors into scaffolds, the stimulatory effects of chondrogenesis and bone regeneration can be promoted, respectively (Raina et al., 2019). In addition, the surface topography of scaffolds can be functionally tailored, from nano-topography to complex micropatterns, providing a range of options to effectively promote cell adhesion and proliferation (Daly et al., 2017). Cartilage defects are often associated with inflammation, and scaffolds with good drug delivery capabilities can also promote cartilage regeneration by carrying drugs or bioactive cytokines that modulate the immune microenvironment (Zhang et al., 2019; Mekinian et al., 2017; Xiong et al., 2022; Xie et al., 2021).

## 3 Different bionic scaffolds for cartilage repair

### 3.1 Natural component-based scaffolds

As natural components present in the ECM, collagen and hyaluronic acid (HA) play important roles in bionic scaffold. These components possess notable biocompatibility and biodegradability, thereby supporting regeneration. Muhonen et al. demonstrated the positive effect of collagen scaffolds on cartilage repair in large animal models (Muhonen et al., 2016). In order to enhance the repair ability, many collagen-based composite scaffolds have been designed. Intini et al. developed innovative collagen scaffolds for cartilage repair, by incorporating type II collagen plus HA into type I collagen scaffold (Intini et al., 2022). Gao et al. developed a type I collagen-HA hydrogel that helped regenerate hyaline cartilage without the need for additional cellular components (Gao et al., 2023). Levinson et al. combined HA-transglutaminase hydrogel with a collagen scaffold for treatment of cartilage defects in an ovine model (Levinson et al., 2021). This combination demonstrated great biocompatibility and facilitated *in situ* cartilage regeneration.

Gelatin is another natural material derived from collagen. Compared to collagen, it does not have an immunogen sequence, so it rarely causes an immune response (Kang and Park, 2021). Due to poor intermolecular interactions, the mechanical property of gelatin does not match that of cartilage, therefore modification or crosslinking with other molecules are of necessity (Sakai et al., 2009). Anand et al. synthesized a crosslinked pullulan-gelatin scaffold, which higher production of cartilage-specific ECM and upregulated sulfated glycosaminoglycan (Anand et al., 2021). Yang et al. reported a gelatin hydrogel modified using alanyl-glutamine (Yang et al., 2022). This modification enables the scaffold to release glutamine through *in vivo* degradation, which, in turn, activates the energy metabolism of chondrocyte. Consequently, this effectively promotes the repair of damaged cartilage.

Derived from natural silk, silk fibroin is widely used for cartilage repair (Silva et al., 2019). It demonstrates good biocompatibility, a slow rate of degradation, and strong mechanical property, which makes it a suitable candidate for cartilage regeneration (Wang et al., 2023). It also maintains chondrocyte phenotype and directs more cartilage-specific protein formation than the collagen-based biomaterials (Bhardwaj et al., 2016).

Chitosan is an analogue of chitin formed by chitin deacetylation. It has potential to become an ideal material in cartilage tissue engineering fields due to its biocompatibility, biodegradability, antibacterial properties, and ability to be molded into various geometries (Muzzarelli, 2009). Currently, some chitosan scaffolds have been used in clinic. Calvo et al. reported a chitosan scaffold combined with microfractures for treatment of patellofemoral osteochondral lesions (Calvo et al., 2021). Poggi et al. reported a chitosan-based scaffold applied in patellar cartilage lesion, which showed acceptable clinical and imaging results at 2 years after implantation (Poggi et al., 2023). Due to the disadvantages of single materials, researchers make further attempts to aggregate multiple materials to construct bionic scaffolds. Yang et al. designed a collagen-gelatin-HA-chondroitin sulfate tetra-copolymer scaffold better than the gelatin scaffold *ex vivo* (Yang et al., 2023). He et al. combined silk fibroin and chitosan to build microsphere scaffold for cartilage reparation (He et al., 2021). Yet the clinical outcomes are still lacking.

### 3.2 Decellularized scaffolds

Decellularized scaffold is obtained from foreign or heterogeneous tissues by removing cells and can be used for seed cell culturing (Zhang et al., 2023) with suitable microenvironment (Li et al., 2023). The advantages of decellularized scaffolds are as follows: First, the risk of inflammation and immune rejection are declined by removing cellular components and antigens (Zhang et al., 2022; Villamil et al., 2020; Giovanni et al., 2019). After decellularization, the microstructure of preserved articular cartilage tissue can provide a high degree of mechanical similarity to native tissue (Luo et al., 2015; Rothrauff et al., 2017a; Rothrauff et al., 2017b; Liu et al., 2025).

A common method for decellularization is freeze/thaw cycle. This physical method can stimulate cell rupture via forming ice crystals (P et al., 2014). However, the ultrastructure of the ECM is disrupted, requiring further removal of cellular debris (Roth et al., 2017).Shen et al. used ultrasound waves to release chondrocytes suitable for cartilage slices no more than 30 μm (Shen et al., 2020). Chen et al. reported a decellularized cartilage from porcine via CO_2_ extraction (Chen et al., 2021). Chemical methods are performed through different acellular chemical reagents. These detergents destroy cell membrane, separating DNA from proteins and removing cellular components from cartilage (Kanda et al., 2023). Schneider et al. developed a protocol via integrating freeze-thaw cycles for devitalization, HA as decellularization agent and the removal of glycosaminoglycans (Schneider and Nürnberger, 2023).

### 3.3 Synthetic polymer scaffolds

Synthesized materials can balance mechanical properties, low immunogenicity, and degradability (Jiann et al., 2023). In cartilage regeneration, poly (ε-caprolactone) (PCL) and poly (lactic-co-glycolic acid) (PLGA) have attracted significant interests.

PCL can be used alone or combined with other polymers to develop scaffold (Chen et al., 2014). When PCL is coupled with the polyethylene glycol (PEG), it is possible to obtain amphiphilic thermosensitive copolymers (PCL-PEG) with shiftable properties upon temperature change (Dethe et al., 2022). Fu et al. designed a PCL-PEG-PCL scaffold which improved cell proliferation and adhesion for cartilage repair (Fu et al., 2016). With the development of 3D printing technique, Li et al. prepared a chitosan hydrogel/3D-printed PCL hybrid with stem cells, hence enhancing the repair of cartilage (Li et al., 2021).

PLGA, a copolymer of polylactide (PLA) and polyglycolide (PGA), has become a widely used material due to its good mechanical properties, non-toxic biodegradation, and controllable biodegradation period (Croll et al., 2004). Xin et al. already proved an electrospun PLGA nanofiber scaffold can promote cartilage differentiation (Xin et al., 2007). However, PLGA has poor hydrophilicity and limited natural cell recognition sites (Wan et al., 2004). Previously, PLGA scaffolds were prepared by electrospinning, while electrospun nanofibers had only one component of the matrix or had a simple structure due to technical limitations. Thus, the application potential of pure synthetic scaffolds is constrained (Zhao et al., 2016). Recently, With the advancement of technology, researchers have designed different forms of PLGA scaffolds for cartilage repair. Through 3D printing, Ding et al. developed a PLGA scaffold with Cell-Free Fat Extract (Ceffe) loaded (Ding et al., 2024). Compared to the pure PLGA scaffold, it showed remarkable vascular formation. Qu et al. developed an open-porous PLGA microspheres as cell carriers for cartilage repair (Qu et al., 2021).

While nano-sized structures exhibit effective simulation of ECM, they may limit cell infiltration (Pham et al., 2006). On the other hand, the construction of micro-nanofibers overcomes this shortcoming and helps to achieve larger pore size, better cell differentiation and ECM construction (Ahmadian et al., 2023). Levorson et al. developed an electrospun scaffold with two different micro-nanofibers, PCL and fibrin, which can maintain scaffold cellularity in serum-free conditions and the deposition of GAGs (Levorson et al., 2013).

### 3.4 Exosome-laden scaffolds

Mesenchymal stem cells (MSCs) have attracted considerable attention in regenerative medicine due to the differentiation potential and immunomodulatory properties (Nikfarjam et al., 2020; Xiaofang et al., 2024). Studies indicated that the pleiotropic effects of MSCs is mediated by paracrine factors (Lai et al., 2015; Rani et al., 2015; Iso et al., 2007). Exosomes, as one of the most important paracrine mediators of MSCs, participate in intercellular crosstalk and alleviate and even reverse the effects of osteoarthritis (Tao et al., 2017; He et al., 2020; Thomas et al., 2023).

Given that exosomes are cleared within a few hours *in vivo*, bionic scaffolds are used as possible vectors for exosome delivery (Chen et al., 2019; Cheng et al., 2023). Pang et al. designed gelatin methacryloyl hydrogels loaded with MSC-derived nanovesicles, which exhibit sustained release and excellent mechanical properties (Pang et al., 2023). They achieved 100% sustained release in 30 days. Shen et al. reported an injectable silk fibrion hydrogel to preserve and release exosomes in a controlled manner, which achieved 85%–89% release in 30 days (Shen et al., 2022). Tao et al. used poly (D,l-lactide)-b-poly (ethylene glycol)-b-poly (D,l-lactide) triblock copolymer gels as carrier of small extracellular vesicles and achieved 80% release in 35 days (Tao et al., 2021).

### 3.5 Gene-activated bioprinted scaffolds

Gene therapy promotes cartilage repair via sustained delivery of therapeutic genes, and approaches have recently been used into clinical trials (Grol, 2024; Muthu et al., 2023). Bionic scaffolds combined with gene complexes are designed to reduce the gene diffusion *in vivo* and control releasing rate *in situ*, thereby ensuring cartilage regeneration (Wang et al., 2024b; Kim and Mikos, 2021).

A variety of scaffolds have been engineered to enhance the efficacy of therapeutic genes. Claudio et al. fabricated a novel microRNA-activated scaffold with composite type II collagen and glycosaminoglycan-binding enhanced transduction system nanoparticles (Intini et al., 2023). This innovative scaffold can improve chondrogenesis. Kim et al. prepared pocket-type micro-carriers with F-127 copolymers and biodegradable PLGA, which promote the chondrogenic differentiation of MSCs (Kim et al., 2021). Electrospun PCL scaffolds loaded silica nanoparticles-associated pDNA were produced by Chernonosova et al. to facilitate successful cell transfection (Chernonosova et al., 2023). Venkatesan et al. used PCL films modified through the grafting of poly (sodium styrene sulfonate) as carriers, in conjunction with a recombinant adeno-associated virus, to facilitate cartilage repair (Venkatesan et al., 2021). Natalia et al. designed HA-based gene-activated cryogel with non-viral vectors based on niosomes to promote *in situ* gene transfection (Carballo et al., 2023). Chen et al. constructed a gelatin methacryloyl (GelMA) hydrogel with seed cells and VEGFa siRNA-LNPs loaded to facilitate cartilage formation (Chen et al., 2022).

## 4 Conclusion and future perspectives

Facing challenges in cartilage repair, the application of biological scaffolds are increasing. With the support of new techniques such as 3D printing and bioprinting, novel scaffolds have been developed to provide strong physical properties for chondrocytes and ECM. Bioactive agents play an important role in cartilage repair including bioactive factors, seed cells, extracellular vesicles (EVs) to promote chondrocyte proliferation and differentiation. Recently, due to the complex structure of cartilage, the construction of multilayered bionic scaffolds has attracted widespread attention (Kolar and Drobnič, 2023; Peng et al., 2023). Some of these multilayered scaffolds have already been used clinically to treat cartilage defects (Boffa et al., 2021; Berta et al., 2020). Cellular behavior is closely related to the *in vivo* microenvironment and endogenous pathways. In order to guide cellular behavior to achieve specific goals, we can mediate different external stimuli such as electricity, light, ultrasound, and magnetism through biomaterials to guide cellular behavior to achieve specific goals. These stimuli-responsive biological scaffold materials have great potential and are also hot spots for future research (Liao et al., 2025).

Bionic scaffolds are expected to provide innovative solutions for the treatment of cartilage injuries. However, there are still several important issues that need to be addressed.

Material performance optimization: In addition to biocompatibility, mechanical properties, and degradability, it is also important to optimize the binding ability of cartilage repair scaffolds to adjacent tissues and reduce the risks associated with rejection and tissue damage.

Bionic structure design: There are multilayered complex structures in normal articular cartilage. Ideal scaffolds should have the ability to replicate the multilayered structure of the articular cartilage. This can be achieved by designing different layers of structures, each exhibiting a corresponding layered function.

Bioactive factors applications: Scaffolds should be designed to control the release speed and targeted delivery of different active ingredients (cytokines, genes, extracellular vesicles, etc.) to optimize their therapeutic potential in cartilage repair.

Cost control and cooperation: In order to make cartilage repair scaffolds more practical, the processability of the scaffolds needs to be improved and the cost of their preparation needs to be reduced. Well-designed clinical trials are needed to facilitate the collaboration between academia, industry, and regulatory agencies to help the scaffolds from laboratory research to clinical use.

In conclusion, the evidence of bionic scaffolds for cartilage repair is evolving, with potential to revolutionize the treatment and have a significant impact on millions of osteoarthritis (OA) patients. While challenges remain, continued research and development of bionic scaffolds will offer attracting potential for the future.
